# Mucoadhesive Polymeric Polyologels Designed for the Treatment of Periodontal and Related Diseases of the Oral Cavity

**DOI:** 10.3390/polym16050589

**Published:** 2024-02-21

**Authors:** Gavin P. Andrews, Thomas Laverty, David S. Jones

**Affiliations:** School of Pharmacy, Queen’s University of Belfast, 97, Lisburn Road, Belfast BT9 7BL, UK; g.andrews@qub.ac.uk (G.P.A.); t.laverty@qub.ac.uk (T.L.)

**Keywords:** polyologel, mucoadhesion, flow rheology, oscillatory analysis, drug release, Raman spectroscopy

## Abstract

The study objective was to design and characterise herein unreported polyologels composed of a range of diol and triol solvents and polyvinyl methyl ether-co-maleic acid (PVM/MA) and, determine their potential suitability for the treatment of periodontal and related diseases in the oral cavity using suitable in vitro methodologies. Polyologel flow and viscoelastic properties were controlled by the choice of solvent and the concentration of polymer. At equivalent polymer concentrations, polyologels prepared with glycerol (a triol) exhibited the greatest elasticity and resistance to deformation. Within the diol solvents (PEG 400, pentane 1,5-diol, propane 1,2-diol, propane 1,3-diol, and ethylene glycol), PEG 400 polyologels possessed the greatest elasticity and resistance to deformation, suggesting the importance of distance of separation between the diol groups. Using Raman spectroscopy bond formation between the polymer carbonyl group and the diol hydroxyl groups was observed. Polyologel mucoadhesion was influenced by viscoelasticity; maximum mucoadhesion was shown by glycerol polyologels at the highest polymer concentration (20% *w*/*w*). Similarly, the choice of solvent and concentration of PVM/MA affected the release of tetracycline from the polyologels. The controlled release of tetracycline for at least 10 h was observed for several polyologels, which, in combination with their excellent mucoadhesion and flow properties, offer possibilities for the clinical use of these systems to treat diseases within the oral cavity.

## 1. Introduction

Organogels are gel networks in which an organic solvent represents the bulk continuous phase [1,2]. Within an organogel, the organic solvent is trapped within a three-dimensional network formed in the presence of a gelling agent within the formulation [3,4]. Typical manufacture of an organogel involves dissolving the gelling agent within a heated organic solvent to produce a liquid solution. Subsequently, when this solution is cooled below the gel transition temperature, a complex viscoelastic gel network is formed [5]. Organogels may be prepared using a wide range of non-aqueous solvents (typically oils and non-polar liquids) and gelation agents, as summarised by Sahoo et al. and Sagiri et al. [1,6]. The choice of solvent and gelation agent directly affects the mechanism by which gelation occurs [7]. Waxes and fatty acids cause gelation through the self-association of fibres or particles of the gelation agent to form a three-dimensional structure of considerable mechanical strength [3,5,8]. These systems require the gelation agent to be solubilised within the solvent by heating and, following cooling, the gelation agent dissociates from the solvent and associates into solid aggregates. Organogels may also be prepared (both in the presence of and absence of water) using a wide range of surface active agents with rheological structuring occurring through micelle formation and entanglement to form a network that entraps the liquid phase [6,9,10,11,12,13]. Examples of other gelation agents have been described by Sagiri et al. and include sterols and derivatives, sugars and derivatives, and Gemini organogelators [6]. Polymers may also be used as gelation agents for organogels. If the gelation is due to non-covalent bond formation between polymer chains, this offers opportunities to design stimulus-responsive organogels [1,3,6,14].

There have been several reported pharmaceutical uses of organogels [1,3,6,15]. These include transdermal drug delivery systems [16,17,18], platforms for controlled/enhanced drug release [19,20], ocular drug delivery [21], vaccine delivery systems [22] and oral drug delivery systems [23,24]. The local (topical) use of organogel platforms has been primarily focused on cosmetic applications [25], transdermal applications (examples provided above) [18], and the treatment of conditions of the skin and nails [26,27,28,29,30,31]. A potential clinical application of organogels is as a platform for the treatment of local disorders of the oral cavity, notably inflammatory disorders, e.g., stomatitis, gingivitis, periodontitis, and lichen planus, and the treatment of infection [32,33]. A wide range of dosage form types has been reported for the treatment of local diseases of the oral cavity, including gels/hydrogels, semi-solid systems, films, fibres, nanoparticles, and microparticles [34,35]. Key to the success of these delivery systems is their retention at the site of application to enable a satisfactory rate of drug delivery for the required period to ensure therapeutic efficacy [36,37,38]. One successful strategy to achieve retention of dosage forms to mucosal surfaces is using mucoadhesive polymers, polymers that chemically interact with mucin and in so doing facilitate retention of the dosage form at the site of application [39]. This strategy has been successfully proven for the improved treatment of periodontal disease [40].

This study uniquely describes the use of novel mucoadhesive non-aqueous gels that have been designed for the treatment of oral disorders. The strategy described herein does not involve the use of traditional oils as the solvent phase but instead uses diol and triol solvents. Considering this, we have termed these systems as polyologels. Polyol solvents are pharmaceutically acceptable, can enhance the solubility of poorly water-soluble drugs, will minimise hydrolytic degradation of therapeutic agents, and do not require the inclusion of preservatives. To enhance the retention of the polyologels at the site of application, we have chosen to include poly(vinyl methyl ether-co-maleic acid, PVM/MA). The mucoadhesive properties of this polymer have been widely reported, including by the authors [36,41,42,43]. Through a comprehensive analysis of the physicochemical properties of these mucoadhesive polyologels, this study will enable the feasibility of these systems as drug-delivery platforms for the treatment of disorders of the oral cavity.

## 2. Materials and Methods

### 2.1. Materials

PVM/MA (Gantrez^®^ SBF97) with an average molecular weight of approximately 1,200,000 Da was kindly donated by ISP, Surrey, UK. The polyol solvents were purchased from Sigma Aldrich, Dorset, UK. All other chemicals were purchased from BDH Laboratory Supplies, Dorset, UK, and were of AnalaR grade or equivalent quality.

### 2.2. Manufacture of PVM/MA Polyologels

PVM/MA (ISP, Surrey, UK) polyologels were manufactured via the slow addition of the appropriate amount of PVM/MA (5%, 10%, 15%, or 20% *w*/*w*) to the chosen mono-solvent (glycerol, pentane-1,5-diol, propane-1,2-diol, propane-1,3-diol, polyethylene glycol (PEG) 400, or ethylene glycol, Sigma Aldrich, Dorset, UK), which was previously heated to 70 °C. Mixing was performed using a Yellow Line mechanical stirrer (Davidson and Hardy, Belfast, UK) until it became homogeneous. After cooling (and if required), tetracycline (1 and 5% *w*/*w*, as the hydrochloride, BDH Laboratory Supplies, Dorset, UK) was added using either mechanical stirring or a palate knife and ointment slab (for higher viscosity systems). Samples were then allowed to equilibrate for 24 h before testing; all testing was completed within 72 h.

### 2.3. Continuous Shear Analysis of Polyologels

Continuous shear analyses of all formulations were performed at 37 °C using a TA system AR2000 rheometer (TA Instruments, Surrey, UK). Flow rheograms were determined using either a 6 cm or 4 cm parallel stainless-steel plate (gap size 1000 µm), the choice of geometry being determined based on sample consistency. Samples to be analysed were applied to the lower plate and allowed fifteen minutes to equilibrate to negate any stresses induced during sample application. The shear stress was applied over a predetermined range, determined by sample consistency, and the rate of shear was determined. In each case, the flow properties of at least five replicates were determined.

The Rheology Advantage software (version 5.8.2) enabled modelling of each polyologel using the Ostwald-de Waele power law model (Equation (1)) and the Cross model (Equation(2)), as follows:

(1) $$\sigma =k{\dot{\gamma }}^{n}$$

where σ refers to the shear stress, $\dot{\gamma }\;$to the rate of shear, k to the consistency, and n is a power law index (indicative of the flow phenotype) [36].

(2) $$\frac{\eta_{o}-\eta }{\eta -\eta_{\infty }}={\left(k\dot{\gamma }\right)}^{m}$$

where η, $\eta_{o},$ and $\eta_{\infty }$ refer to the viscosity, the zero-rate viscosity, and the infinite shear viscosity, respectively; k is the consistency; m is the slope of the curve at the inflexion point; and $\dot{\gamma }\;$to the rate of shear [44].

### 2.4. Oscillatory Analysis of Polyologels

Oscillatory (dynamic) analyses were performed at 37 °C on all formulations using a TA system AR2000 rheometer. Rheological analyses were conducted using either a 6 cm or 4 cm parallel stainless-steel plate (gap size 1000 µm); again, the choice of geometry was determined via sample consistency. Samples to be analysed were applied to the lower plate and allowed fifteen minutes to equilibrate to negate any stresses induced during sample application. For each sample, the LVR (linear viscoelastic region) was determined via a stress sweep at a fixed frequency. The LVR was identified as the region in which the stress and the strain were directly proportional and where the storage modulus (G′) remained constant. Once determined, a frequency sweep from 0.1 to 10 Hz was performed at a strain value selected from within the LVR. The storage modulus (G′), loss modulus (G″), dynamic viscosity (η′), and the loss tangent (tan δ) were then determined using Rheology Advantage software provided by T.A. Instruments. The dynamic rheological properties of at least five replicates were determined.

The relationship between storage modulus and oscillatory frequency was determined using the power law model as follows:

(3) $$G^{\prime }=kf^{n}$$

where G′ is the storage modulus, f is the oscillatory frequency, n is the rheological index, and k is the gel strength.

### 2.5. Mucoadhesion Testing

Mucoadhesion testing was conducted using a TA XT2 Texture Analyser (Stable Micro Systems, Surrey, UK) in adhesion mode as previously described by the authors of [28,29,45]. In brief, 400 mg mucin discs were manufactured using a 13 mm infrared press using a force of 10 tonnes for one minute. The discs produced were then attached to the end of a 10 mm diameter polycarbonate probe via double-sided adhesive tape. Samples to be analysed were transferred into a circular mould with mucoadhesion being determined at 37 °C. All samples were stored in sealed sample vials incubated at 37 °C for 24 h and before testing the disc was pre-wetted with 5% mucin solution with the excess being removed via blotting. A downward force of 0.1N was applied to the polyologel sample and was held for 30 s before being removed at a speed of 10 mm s^−1^. The resultant detachment force (mucoadhesion) of five replicates was determined.

### 2.6. In Vitro Drug Release

In vitro drug release was performed on tetracycline-loaded polyologels using a Caleva 8ST dissolution apparatus. Formulations (2 g) were loaded into circular polycarbonate molds (25 mm diameter, 10 mm height) and placed into the dissolution vessels which contained one litre of phosphate-buffered saline pH 7.4. The temperature within these vessels was maintained at 37 ± 0.5 °C, with the solution being continuously stirred at 25 rev/min using paddles placed 25 ± 2 mm from the surface of the polyologel. Fixed volumes of samples (1 mL) were removed at specific time intervals and filtered through a 0.45 μm syringe filter, with the amount of tetracycline hydrochloride released being analysed through UV spectroscopy (λ_max_ 362 nm) with five replicates being measured for each formulation at each time point. The sample volume removed at each time point was replaced with an equal volume of buffer to maintain the volume within the dissolution vessel. The mass of tetracycline released was calculated using a previously constructed calibration curve, which was linear over the range of 7.5–75.0 µg mL^−1^ (r^2^ = 1.00).

### 2.7. Raman Spectroscopy

Raman spectra were obtained using an Avalon Instruments Raman Station R3 (PerkinElmer Life and Analytical Sciences Inc., Wellesley, MA, USA), with a Class 3B laser emitting at 785 nm. Spectra were recorded from 200–3200 cm^−1^, with a resolution of 2 cm^−1^.

### 2.8. Statistical Analysis

#### 2.8.1. Drug-Free Polyologels

Statistical analysis of the effects of PVM/MA concentration (5, 10, 15, and 20% *w*/*w*) and solvent choice (glycerol, pentane-1,5-diol, propane-1,2-diol, propane-1,3-diol, polyethylene glycol (PEG) 400, and ethylene glycol on zero shear rate viscosity, consistency, rate indices gel strength, rheological index, and crossover frequency of drug-free polyologels were performed using a two-way ANOVA. Similarly, the effect of these factors and oscillatory frequency on formulation viscoelastic properties (i.e., G′, G″, tan *δ*, and η′) were performed using a three-way ANOVA using five representative frequencies (i.e., 1.62, 2.37, 5.39, 7.70, and 9.99 Hz). The statistical analysis of PVM/MA concentration and solvent choice on power Law modelled dynamic moduli (i.e., gel strength and rheological index) were performed using a two-way ANOVA, whereas the effects of PVM/MA concentration and solvent choice on formulation mucoadhesiveness were statistically analysed using a two way ANOVA [36].

#### 2.8.2. Tetracycline-Containing Polyologels

Statistical analysis of the effects of increasing PVM/MA and tetracycline concentration and solvent choice on zero shear rate viscosity, consistency, and rate indices were performed using a three-way ANOVA. Statistical analysis of the effects of increasing the concentration of PVM/MA and tetracycline, solvent choice, and oscillatory frequency on formulation viscoelastic properties (i.e., G′, G″, tan *δ*, and η′) were performed using a four-way ANOVA using five representative frequencies (i.e., 1.62, 2.37, 5.39, 7.70 and 9.99 Hz). Statistical analyses of the effects of increasing the concentration of PVM/MA and tetracycline and solvent choice on Power Law modelled moduli (i.e., *K* and *n*) were performed using a three-way ANOVA. Statistical analyses of the effects of increasing PVM/MA and tetracycline concentration and solvent choice on drug release were performed using a three-way ANOVA, using the time taken for release of 10% and 50% *w*/*w* of total drug loading. Finally, the effects of increasing the concentration of PVM/MA and tetracycline and solvent choice on mucoadhesiveness were statistically analysed using a three-way ANOVA. Post hoc comparisons of means were performed using Tukey’s HSD test, with *p* < 0.05 denoting significance [36].

## 3. Results and Discussion

To be clinically successful, products developed for use in the oral cavity should exhibit certain key properties including, ease of application to and retention at the site of application, prolonged and controlled drug release, and product elasticity (an important factor in drug diffusion through polymer matrices) [40]. Whilst the actual physicochemical properties will change depending on the disease state being treated, the methods used to characterise prototype implants must provide information relevant to potential clinical performance. Hence, flow rheometry provides information relevant to the application of the polyologels to the oral cavity, oscillatory rheometry provides information concerning the rheological structure of the polyologels at equilibrium (which affects drug release), and mucoadhesion analysis provides information relevant to the potential retention of the polyologel at the site of application. Raman spectroscopy was employed to understand the interaction between PVM/MA and the various solvents. In addition, the release of a model antimicrobial agent tetracycline from selected polyologels is presented.

### 3.1. Raman Spectroscopy of Drug-Free Polyologels

**Figure 1:**
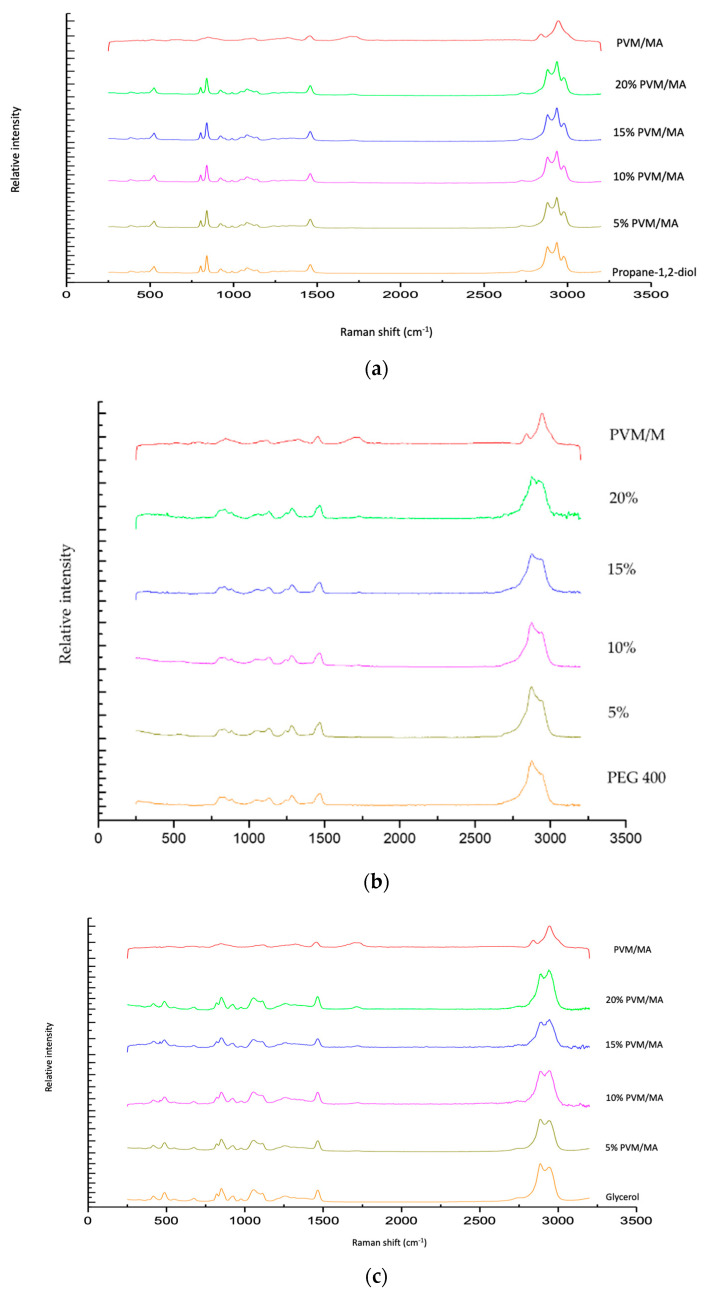
Raman spectra of polyologels composed of propylene 1,2 diol (**a**), PEG 400 (**b**), glycerol (**c**), PVM/MA (5–20% *w*/*w*), and anhydrous PVM/MA (as a control).

**Figure 2:**
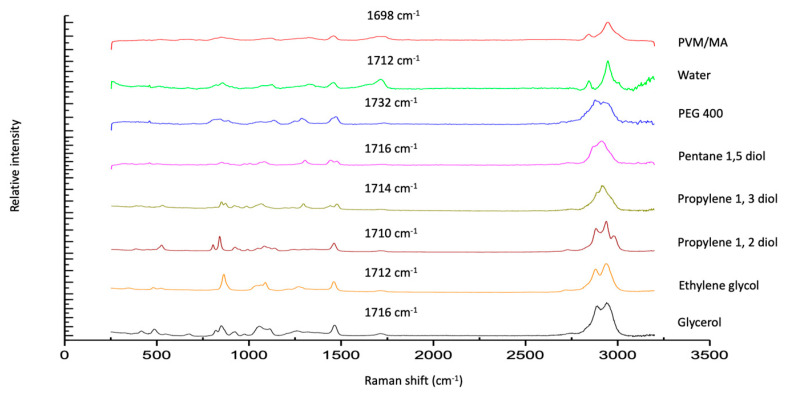
Raman spectra of polyologels composed of diol solvents and PVM/MA (20% *w*/*w*), aqueous PVM/MA (20% *w*/*w*) gels, and anhydrous PVM/MA (as a control).

The Raman spectra of exemplar solvent-PVM/MA polyologels, PVM/MA, and the pure solvents are shown in Figure 1a–c. Most peaks displayed in the spectra may be assigned to the individual solvents; however, a peak at circa 1700 cm^−1^ of weak intensity was observed for anhydrous PVM/MA and its resulting mixtures but was absent within all solvent-only spectra. The addition of water to anhydrous PVM/MA resulted in a small but important shift in the carbonyl band from 1698 cm^−1^ to 1712 cm^−1^. This peak represents the stretching of the carbonyl group which is found only in networks where PVM/MA is present [44]. To examine this further, Figure 2 presents the Raman spectra of the systems containing 20% *w*/*w* PVM/MA with the carboxyl stretch wavenumbers noted. Anhydrous PVM/MA presented a carbonyl band at 1698 cm^−1^, which shifted upon the addition of aqueous or diol/triol solvents to a higher wavenumber. PEG 400 caused the greatest shift in the carbonyl band and may be ascribed to the effects of the large molecular weight of this solvent on the subsequent vibration. Hao et al. [43] reported the interaction of PVM/MA (anhydride) and PVP via Raman spectroscopy and concluded that O-H shifts would typically be a good indication of hydrogen bonding using vibrational spectroscopic techniques; however, the weakness of this band within Raman spectroscopy meant that this was not practical. The authors reported that more valuable information could be obtained on hydrogen bonding from the closer inspection of the stretching of carbonyl groupings and that the small peak shifts observed within the 1600–1800cm^−1^ region were highly suggestive of hydrogen bonding. It may, therefore, be assumed that observed carbonyl shifts in this study are due to interactions of the carbonyl group of PVM/MA and the hydroxyl groups on the various solvents.

### 3.2. Flow Rheometry of Drug-Free and Tetracycline-Containing Polyologels

The flow properties of drug-free polyologels of PVM/MA prepared using different solvents are presented in Table 1 as the consistency and rate index (derived from Equation (1)) and zero rate viscosity (derived from Equation (2)). Increasing PVM/MA concentration significantly increased the consistency and zero rate viscosity and reduced the rate index of all systems under examination. This may be explained by increased intermolecular interaction and polymer entanglement causing high-viscosity networks [46]. The rate index of all polyologels was less than 1, indicative of pseudoplastic behaviour, and is advantageous for the local administration of formulations. The choice of non-aqueous solvent significantly affected the flow rheological properties of the polyologels. The greatest consistencies and zero rate viscosities (independent of PVM/MA concentrations) were exhibited by polyologels prepared using glycerol. Conversely, polyologels prepared using ethylene glycol demonstrated the lowest consistencies and zero rate viscosities, again at all concentrations of PVM/MA. For example, the maximum observed consistency and zero rate viscosity were 2091.3 ± 24.1 Pa.s^n^ and 11,220.00 ± 570.69 Pa.s, observed in the glycerol polyologel containing 20% *w*/*w* PVM/MA. The minimum observed consistency and zero shear rate viscosity were 1.6 ± 0.4 Pa.s^n^ and 1.0 ± 0.0 Pa.s, observed in the ethylene glycol polyologel containing 5% *w*/*w* PVM/MA. Differences in the consistencies and zero rate viscosities of polyologels prepared using the other diols were observed. However, these were dependent on the concentration of PVM/MA, and this accounted for the statistical interaction term between PVM/MA and non-aqueous solvent within the ANOVA. At higher concentrations of PVM/MA (15% and 20% *w*/*w*), greater differences were observed between the consistencies and zero rate viscosities of the polyologel composed of propane 1,2 diol, propane 1,3 diol, pentane 1,5 diol, and PEG 400 but the differences were less marked when the lower concentrations of polymer were used. Most notably, the polyologels prepared using PEG 400 and 15% and 20% *w*/*w* PVM/MA displayed the greatest consistencies and zero shear rate viscosities than those of the other diols. Finally, PVM/MA concentration significantly affected (lowered) the rate index of the polyologels; however, the effects of solvent type on this parameter were less marked.

The effect of the incorporation of tetracycline (as the hydrochloride salt) on the flow rheological properties of polyologels prepared using propane 1,2-diol, PEG 400, and glycerol are shown in Table 2. Visual observation revealed the solubility of tetracycline within the polyologels was dependent on the solvent type and concentration of tetracycline. Tetracycline (1 and 5% *w*/*w*) fully dissolved in Propane 1,2-diol gels and also in glycerol gels but only at the lower drug loading. Tetracycline did not dissolve in polyologels prepared using PEG 400. The incorporation of 1% *w*/*w* tetracycline (as the hydrochloride) did not significantly affect the rheological parameters, whereas further increasing the drug loading to 5% increased the consistency and zero rate viscosity of polyologels composed of 5, 10, and 15 but not 20% *w*/*w* PVM/MA. This disparity (identified as a statistical interaction in the ANOVA) may be, at least in part, due to competition between the drug and polymer for the non-aqueous solvent (in systems where the drug is dissolved) and the effect of suspended drugs on the flow properties. Therapeutic agents have been previously reported to affect the rheological properties of pharmaceutical gels and semi-solids [47,48]. For example, the authors demonstrated the interaction between chlorhexidine and polyacrylic acid in mucoadhesive semisolids and the effects on the rheological properties whenever chlorhexidine was dissolved and dispersed [47], whereas Gadziński et al. reported increased elasticity of gellan-based hydrogels following the incorporation of mesalazine [47]. Conversely, the incorporation of lidocaine into gels of acrylamide/sodium acryloyldimethyl taurate copolymer significantly reduced gel elasticity through screening of the charge on the polymer chains and hence reducing polymer chain expansion through charge repulsion.

### 3.3. Oscillatory Analysis of Drug-Free and Tetracycline-Containing Polyologels

**Figure 3:**
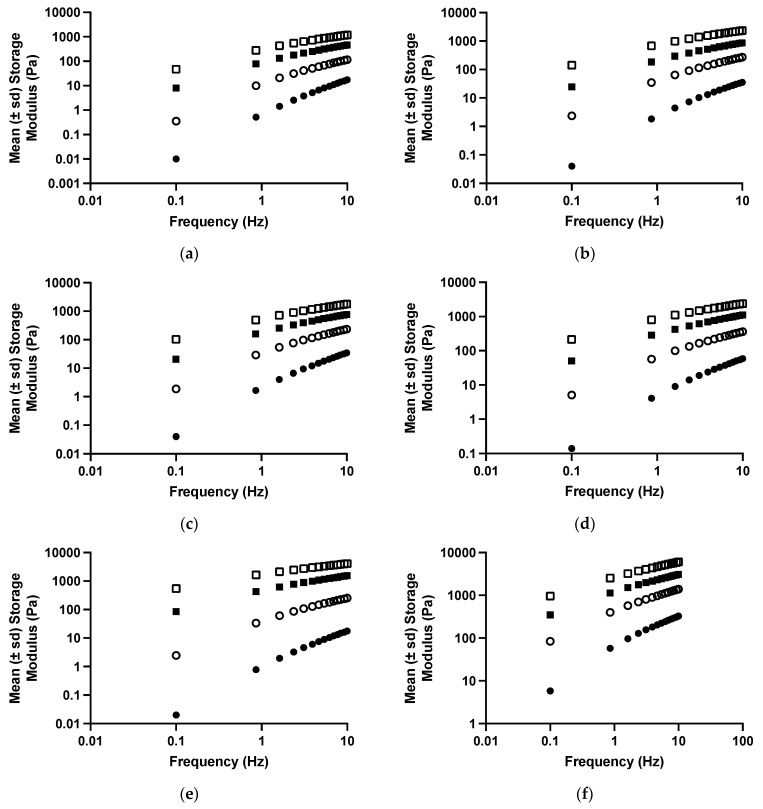
The mean (±s.d.) storage modules of polyologels containing PVM/MA 5% *w*/*w* (closed circles), 10% *w*/*w* (open circles), 15% *w*/*w* (closed squares), and 20% *w*/*w* (open squares), and ethylene glycol (**a**), propane 1,2-diol (**b**), propane 1,3-diol (**c**), pentane 1,5-diol (**d**), PEG 400 (**e**), or glycerol (**f**).

The viscoelastic properties of drug-free polyologels of PVM/MA prepared using different solvents are presented in Figure 3 and Table 3 and Table 4. As the oscillatory frequency increased, the storage modulus (Figure 3) of each polyologel increased, whereas the loss tangent and dynamic viscosity decreased (Table 3). These are characteristic properties of viscoelastic materials [47,49,50,51]. Increasing the concentration of PVM/MA increased the storage modulus and dynamic viscosity and decreased the loss tangent of the polyologels. These observations may be ascribed to greater chain entanglement as the concentration of polymer increased [36]. As was the case with the flow properties, the viscoelastic properties of the polyologels were significantly affected by the choice of solvent; however, more subtle differences in the differential effects of the solvents were identified. In addition, the effect of each solvent on the viscoelastic properties was significantly affected by both the oscillatory frequency and the concentration of PVM/MA. Polyologels prepared with glycerol exhibited the greatest elasticity (largest storage modulus; lowest loss tangent), whereas those prepared using ethylene glycol were the least elastic. Individual differences in the viscoelastic properties of the polyologels prepared using each solvent were observed and may be ascribed to differences in the crosslink density of the gels. To further understand this, the gel strength and exponent were determined by the application of a power law to the relationship between storage modulus and oscillatory frequency (Equation (3)) and, where appropriate, the cross-over frequency (the frequency at which gelation occurs) (Table 5) [52]. The effects of polymer concentration and solvent on gel strength confirmed that glycerol-based polyologels possessed the highest elasticity (based on the greatest observed gel strength and lowest crossover frequency). This study has shown that all polyologels interact with the carboxylic acid group of PVM/MA through the hydroxyl groups (Figure 1 and Figure 2). Glycerol, possessing three hydroxyl groups, enables gels prepared from this solvent to adopt a three-dimensional network structure and is the only solvent within this study to interact in this manner. This would account for the differences in the rheological and viscoelastic properties between glycerol polyologels and the other diol solvents studied in this study. Concerning the other diol solvents, gel elasticity was greater for PEG 400 polyologels than the others. This may be ascribed, at least in part to the flexibility of this polymeric solvent, to optimise its interaction with PVM/MA. Finally, amongst the other diols, increasing the aliphatic chain length (e.g., pentane 1,5-diol) produced polyologels of greater elasticity/gel strength. This is consistent with the hypothesis that this molecular flexibility allows the propensity for a greater number of interactions between this diol and PVM/MA.

The mechanical profile of a gel provides information on the nature of the network and the type of interactions that are involved in its formation [53]. Power Law modelling of the dynamic moduli obtained through oscillatory rheological testing can help in the classification of formulations as gels or sols and may aid in the elucidation of the type of network formed (Table 4). According to the gel classification of Winter and Chambon [54], 20% *w*/*w* PVM/MA in PEG 400, pentane-1,5-diol and glycerol, and 15% *w*/*w* PVM/MA in glycerol are all classified as gel networks (where G′ > G″ and n < 0.5). Power Law indices for all formulation moduli were observed to be greater than zero confirming that the resulting networks were due to physical chain entanglement rather than by covalent cross-linking [55,56].

**Figure 4:**
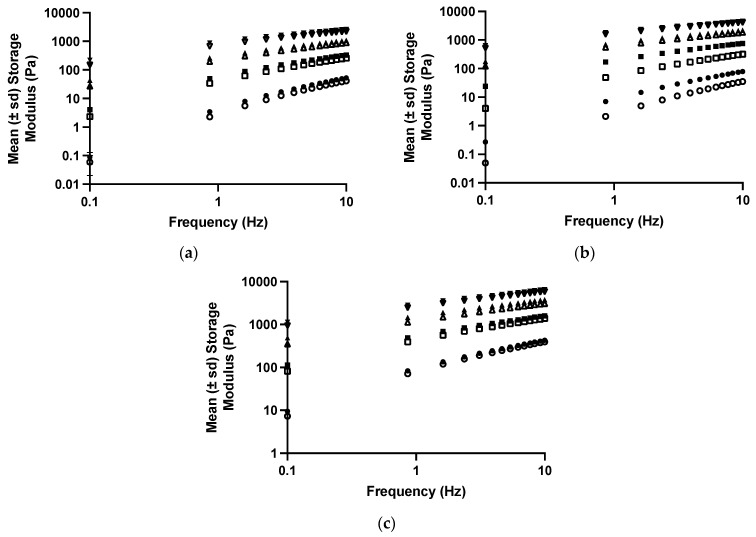
The mean (± s.d.) storage modules of polyologels containing PVM/MA 5% *w*/*w* (circles), 10% *w*/*w* (squares), 15% *w*/*w* (upward triangles), and 20% *w*/*w* (downward triangles). Open symbols refer to polyologels loaded with 1% *w*/*w* tetracycline (as the hydrochloride,) whereas close symbols refer to polyologels loaded with 5% *w*/*w* tetracycline (as the hydrochloride). (**a**–**c**) refer to polyologels prepared using propylene glycol, PEG 400, and glycerol, respectively.

The effect of tetracycline on the viscoelastic properties of polyologels was similar to the effects on the flow properties and illustrated the effect of this dispersed therapeutic agent on these structural properties (Figure 4 and Table 5 and Table 6). Similar effects have been reported by the authors concerning the incorporation of tetracycline [40] and chlorhexidine [57] within aqueous mucoadhesive platforms. As before, all studied networks presented Power Law exponent values which were greater than zero, indicating the presence of physically entangled gel networks, resulting from short-range attractive forces (hydrogen bonding) between polymers [55].

### 3.4. Mucoadhesion of Drug-Free and Tetracycline-Containing Polyologels

The importance of mucoadhesion to the retention of dosage forms within the oral cavity and other mucosal sites has been widely reported, e.g., [40,58]. Formulations for prolonged drug release (and hence enhanced clinical performance) should offer this property and accordingly, the mucoadhesive properties of the polyologels were investigated (Table 4 and Table 6). The concentration of PVM/MA and choice of solvent (but not tetracycline concentration) affected mucoadhesion, defined by the force of detachment between a hydrated mucin disc [36,40]. Whilst there are several mechanisms by which mucoadhesion may occur, it is agreed that polymer chain entanglement between the gel platform and mucin is a key step in ensuring gel retention at the site of application [58]. The study of mucoadhesion has focused primarily on the identification of polymers that offer mucoadhesion (and subsequent structural modifications to enhance this property) and their incorporation into pharmaceutical dosage forms, e.g., pellets/tablets, films/wafers, liquids, pastes, and gels [54]. Furthermore, the study of the mucoadhesive properties of liquids, pastes, and gels has focused on aqueous systems, and hence the study of the mucoadhesive properties of non-aqueous systems has received little attention. In this study, the mucoadhesion of polyologels was dependent upon the concentration of PVM/MA. Increasing PVM/MA concentration increases the number of functional groupings responsible for mucin interaction and bond formation and, following contact with aqueous fluid at the mucosal surface, would provide greater depth of interpenetration into mucin due to greater swelling properties. Solvent choice significantly affected the mucoadhesion, with the most viscoelastic polyologels possessing the greatest mucoadhesion. Glycerol-based PVM/MA polyologels possessed the greatest viscoelasticity and mucoadhesiveness of all studied platforms. The importance of gel viscoelasticity on mucoadhesion has been reported previously, e.g., the authors described the dominant contribution of viscoelasticity of aqueous PVM/MA gels to mucoadhesion [36]. Viscoelasticity, therefore, facilitates mucoadhesion by providing a more elastic network to interact with mucin and to resist cohesive failure. Interestingly, the mucoadhesion of several PVM/MA polyologels could not be measured as these did not possess suitable network structures. It is expected that these polyologels will be rapidly removed from the site of action [59]. It is of interest to compare the mucoadhesive properties of the polyologels described in this study with their aqueous counterparts [36]. Polyologels prepared using glycerol, PEG 400, and pentane 1,5-diol demonstrated significantly greater mucoadhesion than their aqueous PVM/MA counterparts (at identical polymer concentrations). Therefore, the use of polyologels offers greater flexibility in tuning the viscoelastic and mucoadhesive properties for the proposed clinical application. Finally, the incorporation of tetracycline hydrochloride did not affect the mucoadhesive properties of polyologels prepared using propane 1,2-diol, PEG 400, or glycerol (Table 6).

### 3.5. Release of Tetracycline from Polyologels

**Figure 5:**
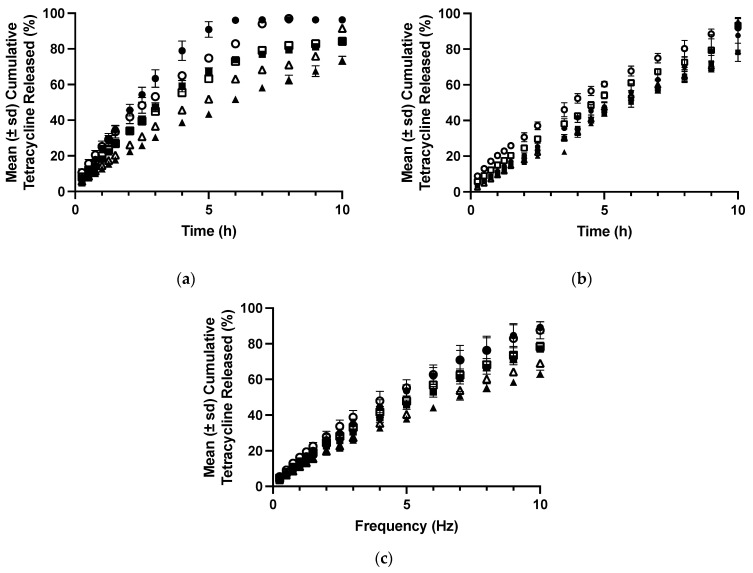
The release of tetracycline from polyologels composed of PVM/MA (10, 15, and 20% *w*/*w*) and propylene glycol (**a**), PEG 400 (**b**), and glycerol (**c**). Symbols: closed and open symbols refer to 1% *w*/*w* and 5% *w*/*w* tetracycline loading (as the hydrochloride), whereas circles, squares, and triangles refer to 10% *w*/*w*, 15% *w*/*w*, and 20% *w*/*w* PVM/MA.

In addition to retention at the site of application, the dosage form must control the necessary release rate of the drug to ensure prolonged clinical efficacy [40]. Therefore, an assessment of the potential suitability of polyologels as a platform for the delivery of therapeutic agents (using tetracycline as a model drug) is presented. The preferred duration of release is dependent on the clinical application, fluid/food intake, and the preferred number of times a dose should be administered. For example, patient compliance would benefit from a daily administration of a dosage form for the treatment of mucosal infection (e.g., candidiasis), whereas in periodontal disease, controlled drug release over some weeks would be preferred [34,40]. The release of tetracycline from the various polyologels was affected by both solvent type and polymer concentration (Figure 5). Specifically increasing PVM/MA concentration reduced the resultant release rate of tetracycline and increased the times required for 10% and 50% of the original drug loading to be released. This may be accredited to the increased elasticity of the polyologel networks. Similarly, the release rate of tetracycline from glycerol polyologels was lower from PEG 400 polyologels and this was again lower compared to propane 1,2-diol polyologels. The effects of both solvent and polymer concentration on drug release may be explained by differences in the viscoelastic properties; polyologels with greater elasticity provide greater control of drug release. The role of gel elasticity in drug release from gels has been previously reported. For example, the authors reported greater control of tetracycline release from mucoadhesive semi-solids [40], whereas Koffi et al. reported the importance of a viscoelastic gel structure to the release rate of quinine [60]. There are further points of interest concerning the release of tetracycline from the polyologels. Firstly, consideration of the release exponent (Table 7) enables an understanding of the mechanism of drug release [61]. In all polyologels, the release exponents (n) were greater than 0.5 but less than 1.0. This confirms that, whilst drug release occurs by diffusion, swelling of the polyologel matrix contributed greatly to the observed release kinetics. Secondly, whilst the release of tetracycline was decreased from polyologels with greater elasticity, the relative magnitude of this is indicative that a second mechanism (swelling) co-contributed to the observed release. Thus, the ingress of aqueous fluid affected the viscoelastic properties of the polyologels and, in so doing, reduced the impact of viscoelasticity on drug release. Finally, the duration of controlled release of tetracycline for several formulations exceeded 10h, with glycerol polyologels showing the lowest rate (and hence highest duration) of tetracycline release. In previous publications, the authors have described the clinical retention of mucoadhesive semi-solid systems within the periodontal pocket [40]. The mucoadhesion of these semi-solids was lower than the polyologels composed of higher concentrations of PVM/MA (15% *w*/*w* and 20% *w*/*w*), and either glycerol or PEG 400 was described in this current study. Therefore, this provides confidence that the current polyologels would be retained within the oral cavity following application. Finally, in this study, the release of tetracycline from the various polyologels was performed under sink conditions, employing a large volume of dissolution fluid (1 L) and stirring with a paddle to understand the release mechanism. These conditions are not representative of those in the oral cavity and, accordingly, the rate of release from the various polyologels would be greater than those expected following application to the oral cavity. For example, whilst the typical daily production of saliva ranges between 0.5 and 1.5 L per day, this is due to a small but constant rate of saliva flow. Furthermore, saliva is eliminated by swallowing ensuring that the volume of saliva in the mouth is low at any time. For example, it has been reported that the flow rate of unstimulated saliva is 0.3–0.4 mL min^−1^; during sleep, it is 0.1 mL min^−1^; however, during mastication, this increases up to 5 mL min^−1^ [62]. The flow of fluid (gingival crevicular fluid) into and out of the periodontal pocket is much lower than the flow of saliva at a few microliters per hour [63].

## 4. Conclusions

This study uniquely describes the rheological, mucoadhesion, and drug-release properties of polyologels composed of a range of diol and triol solvents and polyvinyl methyl ether-co-maleic acid (PVM/MA), designed for the treatment of periodontal and related diseases in the oral cavity. Manipulation of the rheological properties of the polyologels (consistency, flow index, zero shear rate viscosity, storage and loss modulus, loss tangent, dynamic viscosity, gel strength, and rheological index) was performed by the appropriate choice of solvent and by increasing the concentration of polymer. At equivalent polymer concentrations, polyologels prepared with glycerol exhibited the greatest elasticity and resistance to deformation. The opposite was the case with polyologels prepared using ethylene glycol. Within the diol solvents (PEG 400, pentane 1,5-diol, propane 1,2-diol, propane 1,3-diol, and ethylene glycol), PEG 400 polyologels possessed the greatest elasticity and resistance to deformation; the distance of separation between the diol groups play an important role. Evidence of interactions between the carbonyl group on the polymer and the hydroxyl groups of the solvents was provided by Raman spectroscopy of the polyologels. The incorporation of tetracycline affected the rheological properties of polyologels but only at the higher (5% *w*/*w*) concentration and mostly when insoluble in the gel. The mucoadhesive properties of the polyologels were influenced by the viscoelastic properties, with maximum mucoadhesion being shown by glycerol polyologels at the highest concentration of PVM/MA (20% *w*/*w*). Tetracycline did not affect these properties. The choice of solvent and concentration of PVM/MA similarly affected the release of tetracycline from the polyologels, with the lowest rate (and highest prolongation) of release being observed with glycerol polyologels at the highest concentration of PVM/MA (20% *w*/*w*). The mechanism of tetracycline release from the polyologels was a combination of controlling diffusion and swelling, the latter reducing the comparative impact of viscoelasticity on release. The controlled release of tetracycline for at least 10 h was observed for several formulations (PEG 400, Glycerol, 15% *w*/*w*, and 20% *w*/*w* PVM/MA). Based on the observations from this study and previous studies, it is proposed that the polyologels described herein offer a new formulation strategy for the treatment of diseases of the oral cavity, e.g., periodontitis, gingivitis, and candidiasis.

## Figures and Tables

**Table 1:** The effects of solvent type and PVM/MA concentration on the flow rheological properties of polyologels.

PVM/MA Concentration (% *w*/*w*)	Solvent	Ostwald-de Waele Model		Cross Model
		Consistency (Pa.s^n^)	Rate Index	Zero Rate Viscosity (Pa.s)
5	Ethylene Glycol	1.6 ± 0.0	0.8 ± 0.0	1.0 ± 0.0
10		11.3 ± 0.5	0.8 ± 0.0	9.17 ± 0.4
15		84.5 ± 4.6	0.6 ± 0.0	63.5 ± 4.0
20		334.7 ± 15.1	0.4 ± 0.0	204.2 ± 9.5
5	Polyethylene Glycol 400	1.8 ± 0.1	0.84 ± 0.0	1.3 ± 0.1
10		26.8 ± 0.6	0.8 ± 0.0	26.0 ± 0.2
15		335.6 ± 10.6	0.7 ± 0.0	630.0 ± 25.4
20		1310.0 ± 57.4	0.4 ± 0.0	7805.0 ± 159.0
5	Propane 1,2-diol	3.3 ± 0.1	0.8 ± 0.0	2.4 ± 0.
10		27.0 ± 0.6	0.8 ± 0.0	26.6 ± 1.4
15		143.3 ± 6.3	0.7 ± 0.0	202.8 ± 10.9
20		625.2 ± 8.1	0.4 ± 0.0	1077.1 ± 65.2
5	Propane 1,3-diol	2.8 ± 0.1	0.9 ± 0.0	2.0 ± 0.1
10		23.1 ± 1.0	0.8 ± 0.0	21.9 ± 0.36
15		116.9 ± 2.5	0.6 ± 0.0	149.9 ± 8.8
20		481.2 ± 22.1	0.4 ± 0.0	571.1 ± 8.3
5	Pentane 1,5-diol	4.8 ± 0.2	0.8 ± 0.0	3.8 ± 0.2
10		40.4 ± 0.3	0.8 ± 0.0	44.5 ± 0.6
15		188.8 ± 7.8	0.7 ± 0.0	353.6 ± 3.3
20		656.8 ± 7.4	0.4 ± 0.0	2086.0 ± 94.7
5	Glycerol	41.7 ± 0.5	0.82 ± 0.00	49.1 ± 2.8
10		306.4 ± 4.63	0.74 ± 0.00	513.2 ± 6.2
15		903.6 ± 16.3	0.57 ± 0.01	2691.0 ± 76.7
20		2091.33 ± 24.1	0.37 ± 0.00	11,220.0 ± 570.7

**Table 2:** The effects of selected polyol solvents and PVM/MA concentration on the flow rheological properties of tetracycline-containing polyologels.

Solvent	Conc^.^ (% *w*/*w*) Tetracycline	5% *w*/*w* PVM/MA			10% *w*/*w* PVM/MA			15% *w*/*w* PVM/MA			20% *w*/*w* PVM/MA		
		Consistency (Pa.s^n^)	Rate Index	ZRV * (Pa.s)	Consistency (Pa.s^n^)	Rate Index	ZRV * (Pa.s)	Consistency (Pa.s^n^)	Rate Index	ZRV * (Pa.s)	Consistency (Pa.s^n^)	Rate Index	ZRV * (Pa.s)
Propane 1,2-diol	0	3.3 ± 0.1	0.8 ± 0.0	2.4 ± 0.1	27.0 ± 0.3	0.8 ± 0.0	26.6 ± 1.1	143.3 ± 6.3	0.7 ± 0.0	202.8 ± 10.9	625.2 ± 8.1	0.4 ± 0.0	1078.0 ± 65.2
	1	3.3 ± 0.1	0.8 ± 0.0	2.3 ± 0.2	27.4 ± 0.3	0.8 ± 0.0	25.3 ± 1.3	144.3 ± 2.4	0.6 ± 0.0	203.4 ± 7.5	592.9 ± 38.7	0.4 ± 0.0	1065.9 ± 15.8
	5	4.6 ± 0.2	0.8 ± 0.0	3.7 ± 0.0	36.5 ± 0.6	0.8 ± 0.0	37.6 ± 0.6	189.5 ± 2.5	0.6 ± 0.0	322.5 ± 24.3	695.2 ± 14.2	0.4 ± 0.0	1463.8 ± 76.0
PEG 400	0	1.8 ± 0.1	0.8 ± 0.0	1.3 ± 0.0	26.8 ± 0.6	0.8 ± 0.0	25.9 ± 0.2	335.6 ± 10.6	0.7 ± 0.0	630.0 ± 25.4	1310.0 ± 57.4	0.4 ± 0.0	7805.0 ± 158.8
	1	3.4 ± 0.2	0.8 ± 0.0	2.7 ± 0.2	36.6 ± 0.7	0.8 ± 0.0	39.3 ± 1.2	366.5 ± 9.8	0.6 ± 0.0	714.8 ± 36.5	1386.0 ± 28.6	0.4 ± 0.0	7767.8 ± 332.5
	5	7.4 ± 0.3	0.8 ± 0.0	6.9 ± 0.2	116.2 ± 3.3	0.8 ± 0.0	156.0 ± 2.1	507.4 ± 16.3	0.7 ± 0.0	1098.0 ± 57.7	1428.3 ± 67.1	0.4 ± 0.0	7474.8 ± 331.4
Glycerol	0	41.8 ± 0.5	0.8 ± 0.0	49.1 ± 2.8	306.4 ± 4.6	0.7 ± 0.0	513.2 ± 6.2	903.6 ± 16.3	0.6 ± 0.0	2691.0 ± 76.7	2091.3 ± 24.1	0.4 ± 0.0	11,220.0 ± 570.7
	1	42.9 ± 1.3	0.8 ± 0.0	50.5 ± 1.2	295.8 ± 8.2	0.8 ± 0.0	505.4 ± 11.1	904.0 ± 25.2	0.6 ± 0.0	2609.8 ± 22.3	2050.3 ± 41.4	0.4 ± 0.0	11,703.3± 1021.7
	5	59.0 ± 0.2	0.8 ± 0.0	70.3 ± 1.6	396.6 ± 14.2	0.8 ± 0.0	661.2 ± 14.7	1203.7 ± 42.7	0.6 ± 0.0	4347.0 ± 125.9	2075.3 ± 59.6	0.4 ± 0.0	12,330.0 ± 997.0

* Zero-Rate Viscosity.

**Table 3:** Effect of frequency, PVM/MA concentration and solvent type on the loss tangent and dynamic viscosity (*η′)* of polyologels.

PVM/MA% *w*/*w*	Osc.Freq (Hz)	Mean (±s.d.) Loss Tangent						Mean (±s.d.) Dynamic Viscosity (Pa.s)					
		Ethylene Glycol	PEG 400	Propane 1,2 Diol	Propane 1,3 Diol	Pentane 1,5 Diol	Glycerol	Ethylene Glycol	PEG 400	Propane 1,2 Diol	Propane 1,3 Diol	Pentane 1,5 Diol	Glycerol
5	2.37	5.0 ± 0.2	4.3 ± 0.2	3.5 ± 0.0	3.6 ± 0.0	2.7 ± 0.0	1.5 ± 0.0	0.8 ± 0.0	0.9 ± 0.0	1.8 ± 0.1	1.6 ± 0.1	2.6 ± 0.1	12.6 ± 0.7
	5.39	3.2 ± 0.0	3.1 ± 0.0	2.5 ± 0.0	2.6 ± 0.0	2.1 ± 0.1	1.3 ± 0.0	0.7 ± 0.0	0.8 ± 0.0	1.5 ± 0.0	1.3 ± 0.1	2.0 ± 0.1	8.5 ± 0.5
	9.99	2.5 ± 0.0	2.4 ± 0.0	2.0 ± 0.0	2.1 ± 0.0	1.8 ± 0.1	1.2 ± 0.1	0.6 ± 0.0	0.7 ± 0.0	1.2 ± 0.0	1.1 ± 0.1	1.6 ± 0.1	6.3 ± 0.2
10	2.37	2.3 ± 0.1	1.7 ± 0.0	1.7 ± 0.0	1.8 ± 0.0	1.5 ± 0.0	1.0 ± 0.0	4.8 ± 0.3	9.9 ± 0.4	10.4 ± 0.3	8.7 ± 0.2	13.4 ± 0.4	45.2 ± 0.7
	5.39	1.8 ± 0.0	1.4 ± 0.0	1.4 ± 0.1	1.5 ± 0.0	1.3 ± 0.0	0.9 ± 0.0	3.6 ± 0.2	7.0 ± 0.3	7.2 ± 0.2	6.1 ± 0.1	9.1 ± 0.2	28.2 ± 0.5
	9.99	1.5 ± 0.2	1.2 ± 0.1	1.2 ± 0.1	1.3 ± 0.0	1.2 ± 0.0	0.9 ± 0.0	2.8 ± 0.1	5.2 ± 0.4	5.3 ± 0.2	4.3 ± 0.1	6.61 ± 0.10	20.2 ± 0.3
15	2.37	1.4 ± 0.0	1.0 ± 0.0	1.2 ± 0.0	1.2 ± 0.0	1.0 ± 0.0	0.8 ± 0.0	16.2 ± 0.6	48.5 ± 1.8	29.2 ± 1.2	25.8 ± 0.2	35.7 ± 0.7	88.6 ± 3.0
	5.39	1.2 ± 0.2	0.9 ± 0.0	1.0 ± 0.0	1.0 ± 0.0	0.9 ± 0.0	0.7 ± 0.0	10.58 ± 0.43	29.2± 1.1	18.2 ± 0.8	16.1 ± 0.2	21.9 ± 0.5	52.8 ± 2.0
	9.99	1.0 ± 0.0	0.8 ± 0.0	0.9 ± 0.0	0.9 ± 0.0	0.9 ± 0.0	0.7 ± 0.0	7.5 ± 0.4	20.1 ± 0.7	12.6 ± 0.6	11.2 ± 0.1	15.1 ± 0.2	36.9 ± 1.4
20	2.37	1.0 ± 0.0	0.7 ± 0.0	0.9 ± 0.0	0.9 ± 0.0	0.8 ± 0.0	0.6 ± 0.0	38.0 ± 1.4	110.1 ± 5.1	69.8 ± 1.5	54.0 ± 1.5	78.5 ± 0.8	157.8 ± 3.1
	5.39	0.9 ± 0.0	0.7 ± 0.0	0.8 ± 0.0	0.8 ± 0.0	0.7 ± 0.0	0.6 ± 0.0	22.9 ± 0.8	62.9 ± 2.0	40.5 ± 0.7	31.7 ± 1.0	49.6 ± 0.5	93.2 ± 1.6
	9.99	0.8 ± 0.0	0.4 ± 0.0	0.7 ± 0.0	0.7 ± 0.0	0.7 ± 0.0	0.6 ± 0.0	15.3 ± 0.5	42.7 ± 1.1	26.5 ± 0.5	21.1 ± 0.8	36.6 ± 0.4	64.9 ± 1.0

**Table 4:** Effect of PVM/MA concentration and solvent on the gel strength and power law index of polyologels.

Solvent	PVM/MA 10% *w*/*w*				PVM/MA 15% *w*/*w*				PVM/MA 20% *w*/*w*			
	Gel St (Pa)	Power Law Index	Crossover Freq (Hz)	Mucoadhesion (N)	Gel St (Pa)	Rheol Index	Crossover Freq (Hz)	Mucoadhesion (N)	Gel St (Pa)	Rheol Index	Crossover Freq (Hz)	Mucoadhesion (N)
Ethylene Glycol	8.7 ± 0.8	1.2 ± 0.0	Not Observed	Not Observed	73.0 ± 0.5	0.86 ± 0.02	Not Observed	0.2 ± 0.0	270.2 ± 9.0	0.7 ± 0.0	3.0 ± 0.0	0.5 ± 0.0
Propane 1,2-diol	32.1 ± 1.1	1.0 ± 0.0	Not Observed	Not Observed	173.5 ± 7.5	0.8 ± 0.0	5.2 ± 0.0	0.3 ± 0.0	658.1 ± 17.3	0.6 ± 0.0	1.4 ± 0.0	0.7 ± 0.0
Propane 1,3-diol	26.5 ± 1.3	1.0 ± 0.0	Not Observed	Not Observed	148.9 ± 2.8	0.8 ± 0.0	5.8 ± 0.1	0.3 ± 0.0	481.8 ± 14.3	0.6 ± 0.0	1.4 ± 0.0	0.7 ± 0.1
Pentane 1,5-diol	52.9 ± 1.8	0.9 ± 0.0	Not Observed	0.1 ±0.0	271.5 ± 6.4	0.7 ± 0.0	2.6 ± 0.1	0.7 ± 0.0	789.4 ± 7.1	0.5 ± 0.0	0.4 ± 0.0	1.0 ± 0.0
PEG 400	31.3 ± 2.6	1.0 ± 0.0	Not Observed	0.2 ± 0.0	410.8 ± 25.2	0.6 ± 0.1	1.6 ± 0.2	0.8 ± 0.0	1614.4 ± 79.6	0.4 ± 0.0	0.1 ± 0.0	1.2 ± 0.0
Glycerol	384.8 ± 6.7	0.6 ± 0.0	1.7 ± 0.1	0.7 ± 0.0	1117.5 ± 45.4	0.5 ± 0.0	0.2 ± 0.0	1.10 ± 0.0	2539.8 ± 41.4	0.4 ± 0.0	<0.1	1.8 ± 0.1

**Table 5:** Effect of PVM/MA concentration, solvent type, and tetracycline concentration on the gel strength and power law index of polyologels.

Solvent	Conc. (%*w*/*w*) Tetracycline	5% *w*/*w* PVM/MA		10% *w*/*w* PVM/MA		15% *w*/*w* PVM/MA		20% *w*/*w* PVM/MA	
		Gel Strength (Pa)	Power Law Index	Gel Strength (Pa)	Power Law Index	Gel Strength (Pa)	Power Law Index	Gel Strength (Pa)	Power Law Index
Ethylene Glycol	0	0.5 ± 0.0	1.7 ± 0.0	8.7 ± 0.8	1.2 ± 0.0	73.0 ± 0.5	0.9 ± 0.0	270.2 ± 9.0	0.7 ± 0.0
Propane 1,2-diol	0	1.7 ± 0.0	1.4 ± 0.0	32.1 ± 1.1	1.0 ± 0.0	173.50 ± 7.5	0.8 ± 0.0	658.1 ± 17.3	0.6 ± 0.0
	1	1.7 ± 0.1	1.4 ± 0.1	31.1 ± 1.3	1.0 ± 0.0	184.3 ± 4.8	0.7 ± 0.0	650.5 ± 7.5	0.6 ± 0.0
	5	2.4 ± 0.1	1.3 ± 0.0	46.0 ± 0.9	0.9 ± 0.0	251.4 ± 6.6	0.7 ± 0.0	848.9 ± 20.7	0.5 ± 0.0
Propane 1,3-diol	0	1.5 ± 0.0	1.4 ± 0.0	26.5 ± 1.3	1.0 ± 0.0	148.9 ± 2.8	0.7 ± 0.0	481.8 ± 14.3	0.6 ± 0.0
Pentane 1,5-diol	0	3.8 ± 0.2	1.3 ± 0.0	52.9 ± 1.8	0.9 ± 0.0	271.5 ± 6.4	0.7 ± 0.0	789.4 ± 7.1	0.5 ± 0.0
PEG 400	0	0.7 ± 0.1	1.5 ± 0.0	31.3 ± 2.6	1.0 ± 0.0	410.8 ± 25.2	0.6 ± 0.0	1614.3 ± 79.6	0.4 ± 0.0
	1	1.7 ± 0.0	1.4 ± 0.0	44.89 ± 0.7	0.9 ± 0.0	536.5 ± 3.8	0.6 ± 0.0	1575.3 ± 25.9	0.4 ± 0.0
	5	6.43 ± 0.3	1.2 ± 0.0	160.8 ± 3.8	0.7 ± 0.0	691.9 ± 8.5	0.5 ± 0.0	1715.0 ± 138.1	0.4 ± 0.0
Glycerol	0	53.3 ± 3.7	0.8 ± 0.0	384.8 ± 6.7	0.6 ± 0.0	1117.3 ± 45.4	0.5 ± 0.0	2539.3 ± 41.4	0.34 ± 0.0
	1	55.5 ± 2.4	0.8 ± 0.0	378.2 ± 3.9	0.6 ± 0.0	1119.0 ± 18.8	0.5 ± 0.0	2458.1 ± 94.4	0.4 ± 0.0
	5	76.8 ± 2.4	0.8 ± 0.0	472.1 ± 8.	0.6 ± 0.0	1455.4 ± 20.8	0.4 ± 0.0	2608.5 ± 60.9	0.4 ± 0.0

**Table 6:** Effect of PVM/MA concentration, solvent type, and tetracycline (as the hydrochloride) concentration on the loss tangent (at 1 Hz) and mucoadhesion of polyologels.

Solvent	Conc. (%*w*/*w*) Tetracycline	5% *w*/*w* PVM/MA		10% *w*/*w* PVM/MA		15% *w*/*w* PVM/MA		20% *w*/*w* PVM/MA	
		Loss Tangent	Mucoadhesion (N)	Loss Tangent	Mucoadhesion (N)	Loss Tangent	Mucoadhesion (N)	Loss Tangent	Mucoadhesion (N)
Propane 1,2-diol	0	1.9 ± 0.1	Not Observed	1.2 ± 0.1	Not Observed	0.9 ± 0.0	0.3 ± 0.0	0.7 ± 0.01	0.7 ± 0.0
	1	2.0 ± 0.1	Not Observed	1.2 ± 0.0	Not Observed	0.9 ± 0.0	0.4 ± 0.0	0.7 ± 0.0	0.8 ± 0.1
	5	1.8 ± 0.0	Not Observed	1.2 ± 0.0	Not Observed	0.8 ± 0.0	0.3 ± 0.0	0.7 ± 0.0	0.9 ± 0.0
PEG 400	0	2.4 ± 0.0	Not Observed	1.2 ± 0.0	Not Observed	0.8 ± 0.0	0.8 ± 0.0	0.6 ± 0.0	1.2 ± 0.0
	1	2.1 ± 0.0	Not Observed	1.2 ± 0.0	Not Observed	0.8 ± 0.0	0.8 ± 0.0	0.7 ± 0.2	1.2 ± 0.1
	5	1.6 ± 0.0	Not Observed	1.0 ± 0.0	Not Observed	0.8 ± 0.0	0.8 ± 0.0	0.6 ± 0.0	1.2 ± 0.1
Glycerol	0	1.2 ± 0.0	1.3 ± 0.0	0.9 ± 0.0	1.7 ± 0.1	0.7 ± 0.0	1.1 ± 0.0	0.6 ± 0.0	1.8 ± 0.1
	1	1.2 ± 0.0	1.3 ± 0.1	0.9 ± 0.0	1.7 ± 0.1	0.7 ± 0.0	1.1 ± 0.1	0.6 ± 0.0	1.7 ± 0.1
	5	1.1 ± 0.0	1.2 ± 0.1	0.9 ± 0.0	1.8 ± 0.1	0.7 ± 0.0	1.1 ± 0.1	0.6 ± 0.0	1.7 ± 0.1

**Table 7:** The effect of solvent type and PVM/MA concentration on the release of tetracycline (1% *w*/*w* and 5% *w*/*w* loading) from polyologels.

Solvent	Conc. PVM/MA (% *w*/*w*)	Tetracycline 1% *w*/*w* Loading (as the Hydrochloride)			Tetracycline 5% *w*/*w* Loading (as the Hydrochloride)		
		Time for 10% Release, t_10_ (h)	Time for 50% Release, t_50_ (h)	Release Exponent (n)	Time for 10% Release, t_10_ (h)	Time for 50% Release, t_50_ (h)	Release Exponent (n)
Propane 1,2-diol	10	23.2 ± 1.9	142.1 ± 7.3	0.89 ± 0.04	22.9 ± 0.6	154.2 ± 7.4	0.68 ± 0.04
	15	28.7 ± 2.0	197.0 ± 13.1	0.84 ± 0.05	25.2 ± 1.2	214.0 ± 6.8	0.72 ± 0.04
	20	42.8 ± 2.9	363.2 ± 14.6	0.76 ± 0.05	34.2 ± 2.0	281.9 ± 6.5	0.80 ± 0.03
PEG 400	10	50.8 ± 1.6	314.7 ± 9.6	0.89 ± 0.05	20.8 ± 1.5	224.2 ± 5.67	0.68 ± 0.04
	15	53.6 ± 1.0	362.4 ± 10.1	0.88 ± 0.04	33.8 ± 2.2	317.4 ± 12.2	0.79 ± 0.04
	20	57.5 ± 1.5	404.9 ± 18.8	0.92 ± 0.02	64.2 ± 3.5	380.5 ± 12.5	0.89 ± 0.05
Glycerol	10	38.4 ± 1.9	252.0 ± 2.6	0.80 ± 0.04	32.7 ± 2.1	269.7 ± 5.0	0.75 ± 0.05
	15	44.0 ± 1.2	339.0 ± 8.9	0.78 ± 0.04	40.2 ± 2.4	309.5 ± 9.7	0.77 ± 0.04
	20	50.9 ±0.7	437.9 ± 16.2	0.76 ± 0.03	52.2 ± 3.7	389.0 ± 9.1	0.80 ± 0.04

## Data Availability

The data presented in this study are available in the article.
